# Resolving the paradox for protein aggregation diseases: NMR structure and dynamics of the membrane-embedded P56S-MSP causing ALS imply a common mechanism for aggregation-prone proteins to attack membranes

**DOI:** 10.12688/f1000research.2-221.v2

**Published:** 2014-07-22

**Authors:** Haina Qin, Liangzhong Lim, Yuanyuan Wei, Garvita Gupta, Jianxing Song

**Affiliations:** 1 Department of Biological Sciences, Faculty of Science, National University of Singapore, Singapore, 119260, Singapore; 2 NUS Graduate School for Integrative Sciences and Engineering, National University of Singapore, Singapore, 119260, Singapore

## Abstract

Paradoxically, aggregation of specific proteins is characteristic of many human diseases and aging, yet aggregates have increasingly been found to be unnecessary for initiating pathogenesis. Here we determined the NMR topology and dynamics of a helical mutant in a membrane environment transformed from the 125-residue cytosolic all-β MSP domain of vesicle-associated membrane protein-associated protein B (VAPB) by the ALS-causing P56S mutation. Despite its low hydrophobicity, the P56S major sperm protein (MSP) domain becomes largely embedded in the membrane environment with high backbone rigidity. Furthermore it is composed of five helices with amphiphilicity comparable to those of the partly-soluble membrane toxin mellitin and α-synuclein causing Parkinson's disease. Consequently, the mechanism underlying this chameleon transformation becomes clear: by disrupting the specific tertiary interaction network stabilizing the native all-β MSP fold to release previously-locked amphiphilic segments, the P56S mutation acts to convert the classic MSP fold into a membrane-active protein that is fundamentally indistinguishable from mellitin and α-synuclein which are disordered in aqueous solution but spontaneously partition into membrane interfaces driven by hydrogen-bond energetics gained from forming α-helix in the membrane environments. As segments with high amphiphilicity exist in all proteins, our study successfully resolves the paradox by deciphering that the proteins with a higher tendency to aggregate have a stronger potential to partition into membranes through the same mechanism as α-synuclein to initially attack membranes to trigger pathogenesis without needing aggregates. This might represent the common first step for various kinds of aggregated proteins to trigger familiar, sporadic and aging diseases. Therefore the homeostasis of aggregated proteins *in vivo* is the central factor responsible for a variety of human diseases including aging. The number and degree of the membrane attacks by aggregated proteins may act as an endogenous clock to count down the aging process. Consequently, a key approach to fight against them is to develop strategies and agents to maintain or even enhance the functions of the degradation machineries.

## Introduction

Protein aggregation/insolubility is characteristic of a broad spectrum of human diseases, in particular neurodegenerative/aging diseases ^1, 2^, which include Parkinson’s disease (PD), Alzheimer’s disease (AD), Huntington’s disease (HD), spinocerebellar ataxias (SCA), and amyotrophic lateral sclerosis (ALS). In addition, protein aggregation has been shown to play a role in aging ^3^ as well as cardiomyocyte autophagy ^4^ and type II diabetes ^5– 7^. Remarkably, for the above mentioned diseases, aggregation/insolubility of specific proteins can be triggered by either genetic mutations (familiar) or environmental insults (sporadic), which strongly implies that a common mechanism may exist to initiate both familiar and sporadic forms of these clinically distinct diseases. Paradoxically, recent studies have suggested that the accumulation of aggregates is unlikely to be the first step in pathogenesis ^7– 9^. However, the common mechanism to initiate these diseases still remains to be elucidated ^1, 7– 9^.

**Figure 1.**
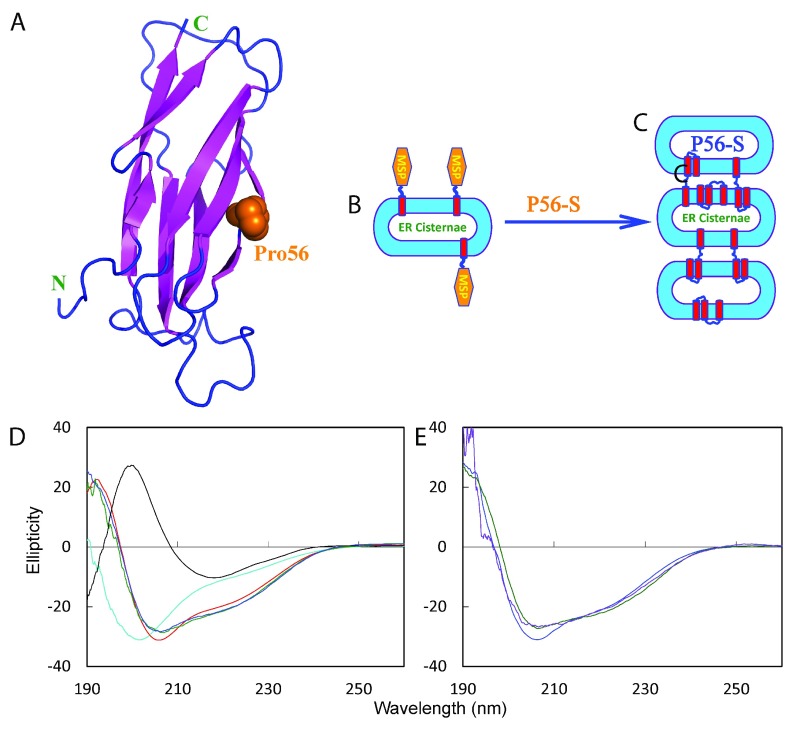
ALS-causing P56S mutation triggers the transformation of the all-β cytosolic MSP domain into a membrane-interacting protein which remodels ER to have stacked cisternae. **A**. 125-residue wild-type MSP domain adopting a seven-stranded immunoglobulin-like β-sandwich fold, with Pro56 displayed in spheres. **B**. The wild-type MSP domain of VAPB is anchored onto the ER membrane facing the cytosol by a C-terminal transmembrane fragment. **C**. The ALS-causing P56S mutant is able to remodel ER to have stacked cisternae by acquiring ability of the P56S MSP to interact with membranes. **D**. Far-UV CD spectrum of the wild-type MSP domain (black), typical of a β structure; and spectra of the P56S MSP in aqueous solution (cyan); in DMPC vesicle (green), bicelle formed by DMPC and DHPC (blue) as well as in DPC micelle (red) at pH 4.0. **E**. Far-UV CD spectra of the P56S MSP in DMPC vesicle (purple), bicelle formed by DMPC and DHPC (green) and in DPC micelle (blue) in 5 mM phosphate buffer at pH 7.5.

ALS is the most prevalent fatal motor neuron disease, yet its underlying mechanism still remains a mystery despite intense studies since the first description more than 130 years ago ^10^. Approximately 10% of ALS cases have a hereditary background, while the other cases are sporadic ^10^. ALS8 was identified from a large Brazilian family, and encodes a mutated P56S major sperm protein MSP domain of VAPB (vesicle-associated membrane protein-associated protein B) ^11^. In the cytosol, the 125-residue MSP domain adopts a seven-stranded immunoglobulin-like β-sandwich fold ( Figure 1A), which is anchored onto the endoplasmic reticulum (ER) surface ( Figure 1B) ^12^. The MSP domain can also be cleaved from its transmembrane anchor to serve as a ligand for the EphA4 receptor ^1, 14^, which is the only-known ALS modifier ^15^. Noticeably, inhibition of EphA4 by a small molecule, called C1, which targets the EphA4 ligand binding channel ^16, 17^ rescued the disease phenotype in ALS models ^15^.

The ALS-causing P56S mutation renders VAPB to form detergent-resistant aggregates *in vivo* upon overexpression ^18^. *In vitro*, we have shown that indeed the P56S MSP mutant is completely insoluble in buffers ^12^. Nevertheless, our unique discovery that all insoluble proteins, including the most hydrophobic integral membrane peptide, could be dissolved in unsalted water and manifest their intrinsic conformations ^19– 22^ allowed us to characterize the residue-specific conformation of the P56S MSP domain in aqueous solution by NMR spectroscopy ^12^. Remarkably, we showed that the P56S mutation is sufficient to completely eliminate its native β-sandwich fold and consequently the P56S MSP domain becomes predominantly-disordered, only with weakly-populated helical conformations over several regions. As such, both *in vivo* and *in vitro* results highlight the association of the aggregation of the P56S mutant with the ALS pathogenesis.

On the other hand, a recent study failed to detect any significant accumulation of aggregates in motor neurons derived from induced pluripotent stem cells of patients carrying the P56S mutation ^23^, suggesting that the accumulation of the P56S VAPB aggregates is not the primary trigger for ALS8 pathogenesis. Furthermore, two recent studies showed that the P56S mutant acquired a novel ability to remodel the endoplasmic reticulum (ER) to have stacked cisternae even without needing the accumulation of aggregates/inclusions ^24, 25^. On the other hand, we discovered that the unstructured P56S, but not wild-type MSP domain, is able to insert into a membrane environment to become a helical structure ^26^, thus providing the underlying mechanism ( Figure 1C) for the observation ^22, 24, 25^.

To shed light on how a point mutation can transform a well-folded, all-β domain into a helical membrane protein, as well as understanding the role of this transformation in initiating ALS pathogenesis, here by solution NMR spectroscopy and paramagnetic relaxation enhancement (PRE), we determined the three-dimensional topology and dynamics of the 125-residue P56S MSP domain in a membrane environment. This represents the first three-dimensional topology of the membrane-embedded helical proteins which are transformed from a well-folded cytosolic all-β domain. Astonishingly, the P56S MSP domain is mostly embedded in the membrane environment with high backbone rigidity, and is composed of five well-formed helices at N- and C-ends linked by a long unstructured loop. Although no membrane-associated fragments could be detected based on hydrophobicity used for identifying classic membrane proteins, the helical residues were found to possess high amphiphilicity that was comparable to those of the membrane-active toxin mellitin and the intrinsically-unstructured α-synuclein that cause Lewy body diseases. This immediately reveals the mechanism for the chameleon transformation: the P56S mutation acts to convert the well-folded cytosolic MSP domain into an unstructured membrane-active protein like mellitin and α-synuclein, by disrupting the specific long-range interaction network that stabilizes the native β-sandwich MSP fold ^12^. Consequently, the previously locked intrinsic amphiphilic and other hydrophobic regions are released and accessible to bulk solvent, which leads to severe aggregation in buffers but, on the other hand, also drives partition into membranes. Since we, and others have extensively shown that insoluble proteins lack tight tertiary packing ^19– 22, 27, 28^; and segments with high intrinsic amphiphilicity universally exist in all proteins including random sequences, regardless of their native structures ^29, 30^, our current study thus resolves the paradox by deciphering that all disease-associated proteins, regardless of being partly-soluble like α-synuclein or insoluble like the P56S MSP, share a common mechanism to attack membranes without needing aggregates. This mechanism might represent the initial step in triggering familiar, sporadic and aging diseases.

## Results

### Formation of the helical conformations in membrane environments

We first accessed the conformational properties in different environments by circular dichroism (CD) spectroscopy. As shown in Figure 1D, the wild-type MSP domain has a far-UV CD spectrum typical of a β-sheet protein. The P56S mutant is predominantly disordered, without any stable secondary structure in aqueous solution, as we previously reported ^12^. Strikingly, the P56S MSP domain transforms into similar helical conformations in 1,2- 1,2-DMPC (dimyristoyl-sn-glycero-3-phosphocholine) vesicles, bicelles formed by DMPC/DHPC (1,2-dihexanoyl-sn-glycero-3-phosphocholine), as well as DPC (n-dodecylphosphocholine) micelles ( Figure 1D). Although the P56S MSP domain gets aggregated immediately in buffers ^12^, once inserted into membranes, it adopts similar helical conformations even in the presence of phosphate buffer at pH 7.5 ( Figure 1E).

**Figure 2.**
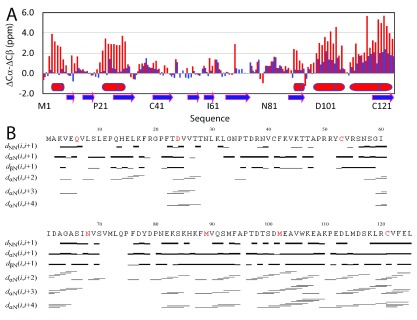
NMR evidence for well-formed helices of the P56S MSP upon partitioning into DPC micelles. **A**. Residue specific (ΔCα-ΔCβ) values of the P56S MSP in aqueous solution (blue) and in DPC micelle (red). The blue arrows are used for indicating the β-strands in the wild-type MSP structures and red cylinders for helices formed in DPC micelles. **B**. NOE connectivities defining secondary structures of the P56S MSP in DPC micelle. The seven residues selected for spin-labeling are colored in red.

By extensively screening lipid component, solution and temperature conditions for NMR experiments, we succeeded in acquiring and subsequently analysing a large set of high-quality NMR spectra in DPC micelles. While consistent with CD data, the NMR chemical shift index ^31, 32^ demonstrates that in aqueous solutions the P56S MSP domain is highly unstructured, and only has weakly-populated helical conformations over several regions. Upon partitioning into the membrane environment, five regions have very large (ΔCα-ΔCβ) chemical shifts comparable to those expected for the well-formed helix, unambiguously showing the formations of stable helices over Lys3-Val7, Phe22-Leu30, Val90-Met93, Asp98-Lys107 and Asp116-Leu125 ( Figure 2A). On the other hand, there is no region retaining the native β-sheet secondary structure. The formation of the helices is further supported by the extensive manifestation of NOEs defining the helical structure, which include d _NN(i, i+1)_, d _αN(i, i+2)_, d _αN(i, i+3)_, and d _αN(i, i+4)_ ( Figure 2D). Amazingly, there is a long region over residues Gly33-His86 without significant changes of chemical shifts upon partitioning into the DPC micelle, indicating that even in the membrane environment this region remains largely unstructured as in aqueous solution.

### Three-dimensional topology of the P56S MSP in a membrane environment

By analyzing ^15^N- and ^13^C-edited NOESY spectra of the P56S MSP domain in both DPC and deuterated DPC, we identified a large set of NOEs defining the α-helices but only very limited long-range NOEs. Thus, to define its three-dimensional topology, we introduced the free radical probe, MTSSL at seven sites as indicated in Figure 2B. Subsequently we utilize paramagnetic relaxation enhancement (PRE) to obtain long distance constraints by the well-established approach ^33– 35^. Finally using X-PLOR and CNS ^36, 37^, we calculated the three-dimensional topology of the P56S MSP domain in a DPC micelle with experimental constraints including distances derived from 339 sequential, 162 medium-range and 7 long-range NOEs; and 465 PREs, as well as 59 pairs of phi and psi dihedral angles predicted by TALOS ( http://spin.niddk.nih.gov/bax/nmrserver/talos/) ^38^.

**Figure 3.**
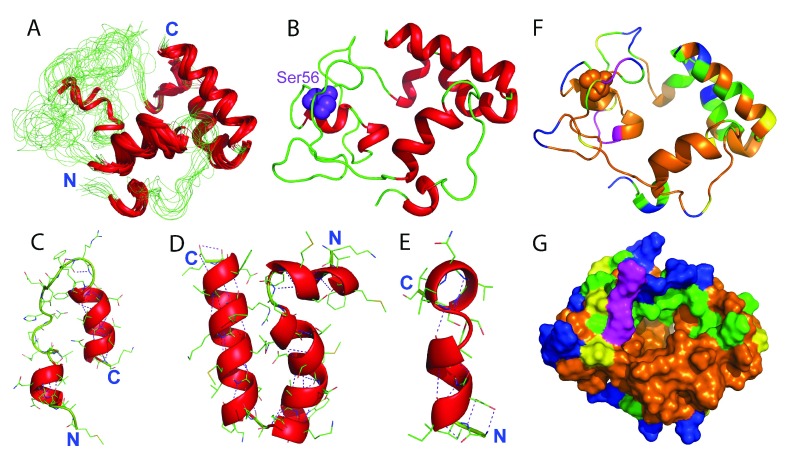
Three-dimensional structure of the P56S MSP in DPC micelles. **A**. Superimposition of the 10 selected NMR structures of the P56S MSP in DPC micelles. **B**. The lowest-energy structure. **C**. Two helices formed at the N-terminus. **D**. Three helices formed at the C-terminus; and **E**. Helices formed in the middle only found in two NMR structures. The purple dashed lines are used to indicate hydrogen bonds. **F**. Ribbon and **G**. surface representations of the P56S MSP in DPC micelle with the display of residues accessible to Mn2+ (green), to gadodiamide (blue). Yellow is used to color Pro residues and purple for residues with missing HSQC peaks.

Figure 3A presents the superimposition of the 10 lowest-energy structures which are composed of five well-formed helices over residues 3–7, 22–30, 90–94, 98–107 and 116–125, consistent with NMR chemical shifts and NOE patterns ( Figure 2). The long region over residues Gly33-His86 has no well-formed secondary structure, only with helices over Ile61-Val71 in two structures. The P56S mutation is located in the unstructured loop ( Figure 3B). In all 10 structures, the orientation among the five helices is well-defined, with average RMS deviations of 1.9 Å for all atoms; 1.6 Å for heavy atoms and 0.9 Å for backbone atoms if only superimposed over the five helices. This indicates that the incorporation of PRE-derived long-range distances into the structure calculation is indeed a very effective approach to define the overall topology, as extensively demonstrated ^33– 35^. Noticeably, backbone hydrogen bonds are extensively formed within the helices ( Figure 3C, 3D and 3E). This observation supports the previous notion that as in membrane environments, proteins are significantly shielded from the water molecules which have strong capacity to form intermolecular hydrogen bonds with protein atoms, proteins thus acquire strong ability to form intra-hydrogen bonds, thus favouring the formation of helix secondary structures. This so called “hydrogen-bond energetic” in fact represents a main force to drive the partition of amphiphilic proteins like mellitin into membranes ^39– 42^.

Unlike classic membrane proteins, no tight tertiary packing exists in the membrane-embedded P56S MSP domain, most likely due to the fact that it is transformed from a cytosolic all-β protein and therefore owns no specific tertiary interactions acquired in evolution for the classic membrane proteins. As a consequence, it represents a nice example of a protein in which folding can indeed stop in the middle of the stepwise folding models, namely at the formation of secondary structures ^40, 43^, thus highlighting the indispensable role of specific long-range interactions in specifying the tertiary structure of membrane proteins. The loose tertiary packing can in fact offer an advantage to rearrange the tertiary topology but to retain very similar secondary structures ( Figure 1E) in different membranes as different lipids have been shown to poses no significant effects on the formation of the helix ^44^. In fact, some of such non-classic properties such as presence of unstructured loops within membranes are starting to be observed even in classic membrane proteins ^45^.

We also used HSQC titrations with two paramagnetic agents, gadodiamide and Mn ^2+^, to probe the exposure of the P56S MSP domain in the DPC micelle ^35^. Interestingly, 17 backbone amide protons are accessible to gadodiamide ( Figure 3F), indicating that only a small portion of residues are exposed to bulk water and therefore the P56S MSP is mostly embedded in the membrane environment. Furthermore, 30 extra backbone amide protons are accessible to Mn ^2+^, suggesting that these residues are located in the polar head-group phase of DPC micelle. As such, ~60% residues are possibly buried in the non-polar hydrocarbon phase, or/and involved in forming hydrogen bonds, which include the N-terminal second helix over residues Phe22-Leu30 and a large portion of unstructured loop ( Figure 3F and 3G).

### Backbone dynamic properties of the P56S MSP domain in a membrane environment

**Figure 4.**
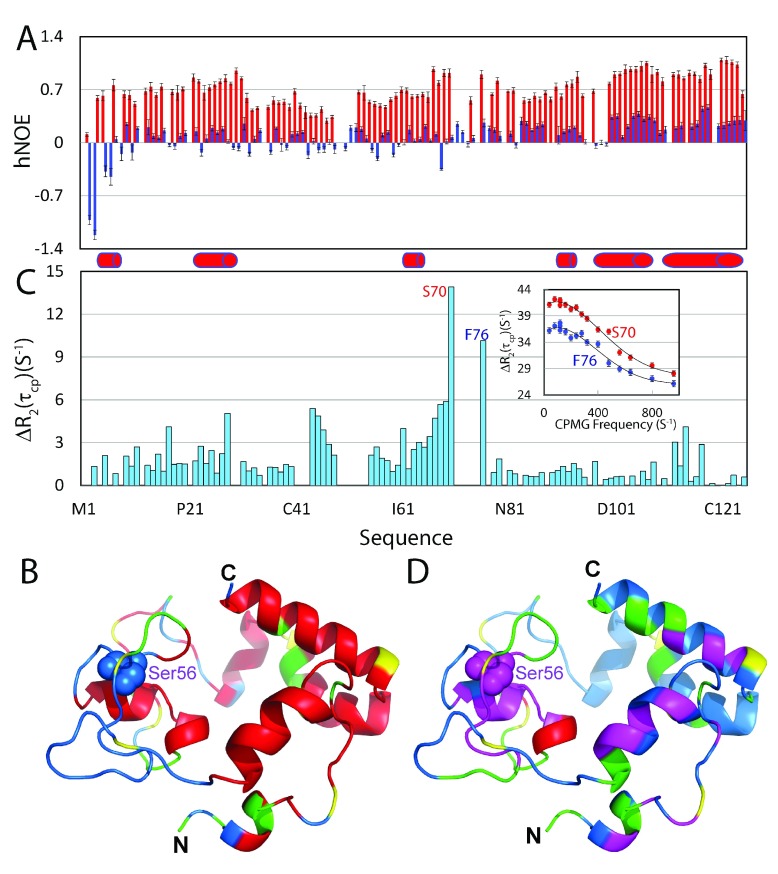
Backbone dynamics on the ps-ns and µs-ms time scales. **A**. { ^1^H}– ^15^N heteronuclear steady-state NOE (hNOE) of the P56S MSP in aqueous solution (blue) and in a DPC micelle (red). **B**. Structure of the P56S MSP in DPC micelles, with Pro residues colored in yellow; missing or overlapped residues in green and residues with hNOEs > the average in red. **C**. Difference of effective transverse relaxation rate R _2_(τ _cp_) at 80 and 960 Hz. Inlet: dispersion curves for two residues S70 and F76. Red cylinders are used to indicate helices formed in DPC micelles. **D**. Structure of the P56S MSP in a DPC micelle, with Pro residues colored in yellow; residues missing or with data having large noise in green; and residues with ΔR _2_(τ _cp_) > 2 in pink and residues with ΔR _2_(τ _cp_) > 6 in red.

To pinpoint the backbone dynamic properties of the P56S MSP in aqueous solution and in the membrane environment, we acquired the heteronuclear NOE which reflects the backbone motions on the ps-ns time scale ^20, 46– 48^. In aqueous solution, very small hNOEs were observed on the P56S MSP residues with an average of 0.08, and several N-terminal residues even had negative hNOE ( Figure 4A and 4B), indicating that the P56S MSP residues are very flexible in aqueous solution. In contrast, once embedded in the membrane environment, all P56S MSP residues have positive hNOE values, with an average of 0.71. In particular, the C-terminal residues forming the helix had hNOEs reaching 1, which was comparable to those observed on any well-folded proteins ^46– 48^. Strikingly, although in general residues forming helices have larger hNOEs, the unstructured regions over Gly33-His86 also have hNOEs much larger than the corresponding residues in aqueous solution, with an average of 0.6. This observation is consistent with the titration results by gadodiamide and Mn ^2+^ that the majority of the P56S MSP residues are embedded in membrane environment, which thus have highly restricted backbone motions on the ps-ns time scale, even without any regular secondary structure.

We further used ^15^N CPMG relaxation dispersion experiments to assess the backbone motions on the µs-ms time scale ^49^. While the P56S MSP in aqueous solution had no detectable backbone motions on the µs-ms time scale (data not shown), many P56S MSP residues had backbone motions on the µs-ms time scale in the membrane environment ( Figure 4C and 4D). In particular, significant conformational exchanges could be observed over residues Val44-Thr47 and Ser66-Phe76. The disappearance of HSQC peaks for residues Val71-Gln74 is likely due to large conformational exchanges on the µs-ms time scale. Unfortunately we have collected the CPMG relaxation dispersion data at 500 MHz but the quality is very poor. As such, only based on the data at one field (800 MHz), we were not able to fit the data to obtain parameters for the conformational exchanges. As many important biological events occur on the µs-ms time scale, the existence of significant µs-ms motions in the P56S MSP domain might impose considerable perturbations/damage to the biological functions of the membranes.

## Discussion

All living cells and organelles in eukaryotic cells are surrounded by biological membranes. Most biological membranes are not only composed of phospholipids, but contain a large fraction of proteins embedded within the lipids. This protein fraction is estimated to make up half of the mass of a biological membrane ^50^. Membrane proteins play various key roles in essential biological processes including cell signalling, transport of membrane-impermeable molecules and intercellular communication. Consequently, membrane proteins constitute the largest class of drug targets ^51^. These classic membrane proteins have a high hydrophobicity, which plays a predominant role in their membrane-insertion, folding and stabilization ^52, 53^. By calculating hydrophobicity, a genome-wide analysis revealed that 20–30% of the open reading frames (ORFs) of various genomes encode integral helix-bundle membrane proteins ^54^.

**Figure 5.**
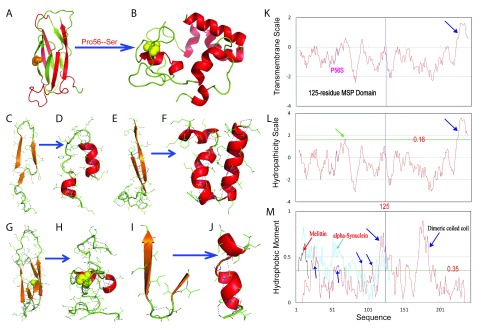
Chameleon transformation from the all-β cytosolic fold into a membrane-embedded helical protein. **A**– **B**. Chameleon transformation from a seven-stranded immunoglobulin-like β-sandwich fold to a membrane-embedded helical structure. **C**– **J**. Secondary structures of different regions adopted in the wild-type and membrane-embedded P56S MSP respectively. **K**. Transmembrane scale of the full-length wild-type (blue) and P56S (red) VAPB (243 residues) calculated with the previous method ^56^. **L**. Hydrophobicity scale calculated with the previous method ^55^. **M**. Hydrophobic moment of the full-length wild-type (blue) and P56S (red) VAPB (243 residues) calculated with the previous method ^57^. Hydrophobic moment of the honeybee membrane toxin mellitin is colored in black and that for α-synuclein in cyan.

**Figure 6.**
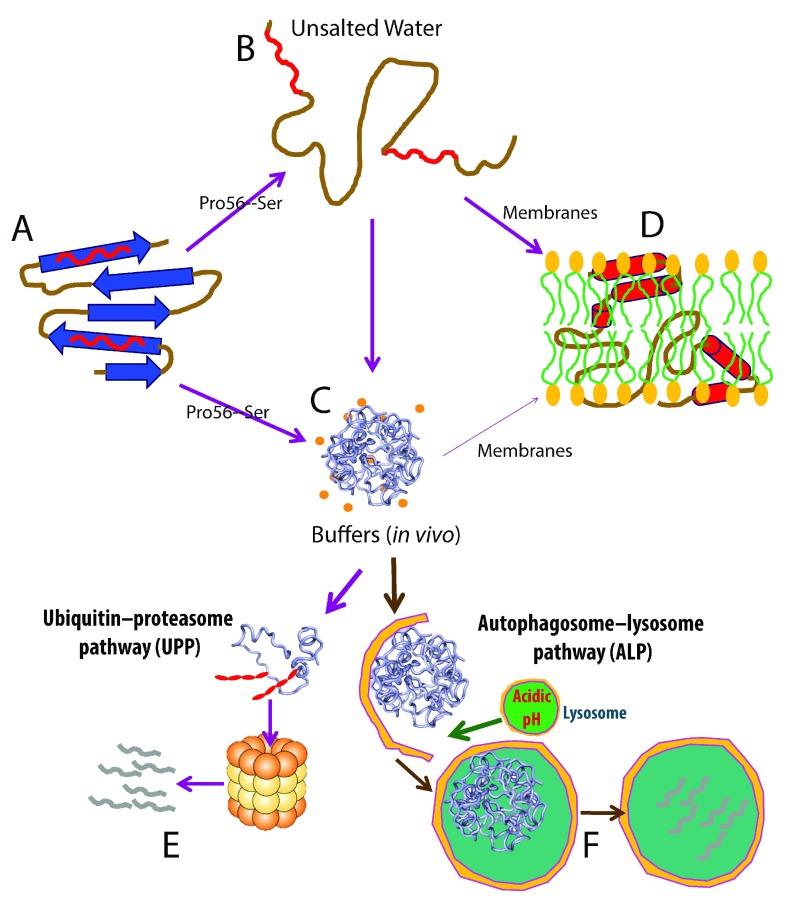
Mechanism for the chameleon transformation and partition into membranes. **A**. The wild-type protein domain like MSP adopts a well-folded three-dimensional structure and therefore its intrinsic amphiphilic and other hydrophobic patches are locked and shielded from being accessible to bulk solvent, thus being highly-soluble in salted aqueous solution. **B**. Some mutations on a well-folded protein like the ALS-causing P56S one are sufficient to completely eliminate its native fold. This results in improper exposure of the intrinsic amphiphilic and other hydrophobic patches. The protein then becomes only soluble but unstructured in unsalted aqueous solution, but is aggregated *in vivo* with ~150 mM ion concentrations ( **C**). **D**. The unstructured mutant acquires the ability to spontaneously partitions into membranes driven by hydrogen bond energetics resulting from forming an amphiphilic helix. As we have shown that insoluble proteins are only insoluble in buffers but soluble in unsalted aqueous solution, even the aggregated mutant is able to partition into the membrane upon having access to membranes under some conditions. However, under normal physiological conditions, aggregates may be immediately detected and subsequently removed by different degradation machineries including ubiquitin-proteasome ( **E**) or/and autophagosome-lysosome ( **F**) pathways. However, once these machineries become dysfunctional due to aging, or/and are inhibited by abnormal conditions, which are generally found to trigger neurodegenerative diseases, aggregates may get become accumulated, which may increase the chance of them accessing/attacking membranes.

Here, we determined the three-dimensional topology of a membrane-embedded helical protein which is transformed from a cytosolic all-β domain, triggered by an ALS-causing P56S mutation ( Figure 5A and 5B). Unexpectedly, five well-formed helices in the membrane environment adopted β-strands in the native MSP fold ( Figure 5C–5J). Based on the hydrophobicity ^55, 56^, which accurately predicts the transmembrane helix at the VAPB C-terminus, no membrane-associated helix can be detected within either wild-type or P56S MSP domain ( Figure 5K and 5L). Nonetheless, calculation of the hydrophobic moment ^41, 57^ revealed that the helical residues have high amphiphilic α-helix potential ^29, 30^, which include Phe22-Leu32 and Cys53-Ala63, Asn68-Val69, Ser92, Lys107 and Asp116-Leu125 ( Figure 5M). In particular, two regions over Phe22-Leu32 and Asp116-Leu125 have amphiphilicity comparable to mellitin, a honeybee membrane-active toxin ^39, 58– 60^, and the intrinsically-unstructured α-synuclein that triggers Parkinson’s disease ^61– 65^. Both of them are unstructured in an aqueous solution, and have a high tendency to aggregate, but spontaneously insert into membranes to form amphiphilic α-helices. Therefore, the mechanism for the chameleon transformation becomes clear: by eliminating the well-folded all-β MSP fold to release previously locked amphiphilic and other hydrophobic patches ( Figure 6A), the P56S mutation acts to convert the cytosolic MSP domain into a mellitin- and α-synuclein-like membrane-active protein ( Figure 6B), which has an even higher tendency to aggregate in buffers ( Figure 6C), but shares the potential to partition into membrane interfaces ( Figure 6D) driven by hydrogen-bond energetics resulting from forming helix ^39– 42^. Therefore the insoluble P56S MSP is fundamentally indistinguishable from partly-soluble mellitin and α-synuclein, designated here as “dynamic membrane proteins”, but it is significantly different from classic membrane proteins in two aspects: 1) it has lower hydrophobicity and therefore amphiphilicity is expected to considerably contribute to its insertion, folding and stabilization in the membrane environments, and 2) it lacks the tight tertiary packing which may thus allow partitioning into different membranes by rearranging its tertiary topology, but retaining the similar secondary structures. The “dynamic membrane proteins” exemplified by mellitin and α-synuclein are extensively characteristic of these non-classic properties. Early studies of the bacterio-rhodopsin structure suggested that membrane proteins are “inside-out”. In other words, they consist of a hydrophilic interior and a hydrophobic exterior ^66^. However, further studies indicate that this rule is not generally applicable even to classic membrane proteins ^40, 66– 68^. Also there appears to have no major driving force to bury polar residues within the protein interior ^66^. The energetic cost for inserting polar groups into a lipid environment is not that high if based on the biological hydrophobicity scale, rather than on other hydrophobicity scales, most of which were derived by utilizing apolar solvents ^40, 66– 68^.

Systematic studies disclosed a surprising fact. Segments with high amphiphilicity exist in all proteins, including randomly-generated sequences regardless of their native structures ^29, 30^. Indeed, nature has exploited a variety of polypeptides, including mellitin, with high amphiphilicity, to achieve antimicrobial, antifungal, antiviral, or anticancer activities by attacking biological membranes to modulate the structural and dynamical properties of the lipids on different length- and time scales ^69^. Interestingly, partly-soluble proteins, causing various human diseases, have also been shown to attack membranes by transforming their unstructured states in aqueous solution, to amphiphilic helixes in membranes. These proteins include: prions of spongiform transmissible encephalopathies ^70, 71^, amyloid beta-(1–40) and beta-(1–42) peptides of Alzheimer’s disease ^72, 73^, tau tangles of Alzheimer’s disease ^74^, α-synuclein of Parkinson’s disease ^62, 64, 65^, huntingtin of Huntington’s disease ^75– 77^ and the islet amyloid polypeptide of type II diabetes ^5, 7^.

Facilitated by our unique discovery that previously-thought insoluble proteins are only buffer-insoluble but in fact soluble in unsalted aqueous solution ^19– 22^, we have recently discovered that all of the insoluble proteins we tested were able to interact with membranes to different degrees ^22^. Here, the determination of secondary structures and three-dimensional topology of the buffer-insoluble P56S MSP mutant in a membrane environment showed that it is fundamentally indistinguishable from partly-soluble α-synuclein and other disease-causing aggregated proteins. Furthermore, we also found that although the ALS-causing T46I mutation does not eliminate, but only destabilizes the MSP fold. However, the T46I mutant appears to also have amphiphilic and other hydrophobic patches more accessible to bulk solvent than the wild-type MSP, thus leading to aggregation in buffers as well as transformation into a helical conformation like the P56S MSP at high DPC concentrations ^14, 22^. Therefore, together with previous results, our present study establishes that aggregated proteins causing diseases, regardless of being partly-soluble or insoluble in buffers, share a common mechanism to initially attack membranes without the need to form aggregates. An interesting question thus arises if all proteins contain amphiphilic segments, why are aggregated proteins closely associated with various human diseases? Firstly, for well-folded proteins like the wild-type MSP domain, their surface residues are hydrophilic while hydrophobic/amphiphilic segments are locked inside, thus inaccessible to interacting with membranes. Secondly, for partially-folded or unfolded proteins, our results from characterizing insoluble proteins reveal that insolubility/aggregation in buffers is mostly due to the improper exposure of hydrophobic patches including those in amphiphilic regions. Therefore, the high tendency of a protein to aggregate reflects that it has highly-accessible hydrophobic or/and amphiphilic patches, which are also driving forces to partition it into membrane interfaces. In other words, the factors for driving aggregation in buffers and partitioning into membranes are at least partly overlapped. Consequently the paradox is resolved: proteins with a higher tendency to aggregate have stronger intrinsic potential to partition into membranes but the formation/accumulation of aggregates is not a prerequisite for this initial interaction with membranes. The accumulation of these proteins in membranes will lead to the formation of channel/aggregates/amyloid fibrils as previously proposed ^7, 8, 69, 78– 84^.

The ability of aggregated proteins to strongly interact with membranes implies that their primary/first step to initiate pathogenesis might be to modulate the structural and dynamical properties of the lipids by a variety of mechanisms that have already been proposed ^7, 8, 69, 78– 84^. As implied from our results here, the numbers of dynamic membrane proteins in cells are much larger than previously recognized. Cellular membranes may therefore be under constant attacking by these proteins, thus rationalizing the observation that most aggregation causing diseases are neurodegenerative diseases and aging as neurons such as cortical neurons are rarely replaced ^85^. Once they get damaged, serious phenotypes will manifest. This may also explain why plants have no aging. Further formation/accumulation of aggregates may radically impose physical stresses/damages to membranes as well as on whole cells, which may be required for pathogenesis of some diseases. As a consequence, the tissue-specific expression of aggregated proteins may be one main factor in manifesting disease phenotypes. On the other hand, the wild-type proteins as exemplified by VAPB, whose mutants become aggregated and cause diseases, physiologically functions as enzymes or signalling components. As a result, the loss of these functions due to mutations may lead to disease-specific phenotypes as observed for the VAPB-MSP domain ^12– 15^. Probably, unlike the P56S VAPB mutant which can be delivered to the ER without requiring the formation of aggregates due to the presence of the C-terminal ER-anchoring helix ^24, 25^, most cytosolic insoluble mutants will not be able to access membranes under normal physiological conditions as they get aggregated immediately after synthesis and subsequently degraded by complex machineries ( Figure 6E and 6F). Moreover, it also appears challenging to detect the initial interaction between these cytosolic aggregated proteins with membranes at the early stage because most of them may not cause significant morphological changes in cells. Only upon proteasomal inhibition, a condition commonly found in neurodegenerative diseases ^86^, do aggregated proteins such as VAPB3, an insoluble splicing variant of VAPB without an ER-anchored region, accumulate and thus have opportunities to access and attack membranes, and thus lead to sporadic diseases. Indeed, an increased expression of the wild-type α-synuclein due to gene duplication and triplication is required to initiate Parkinson’s disease ^61, 63^.

**Figure 7.**
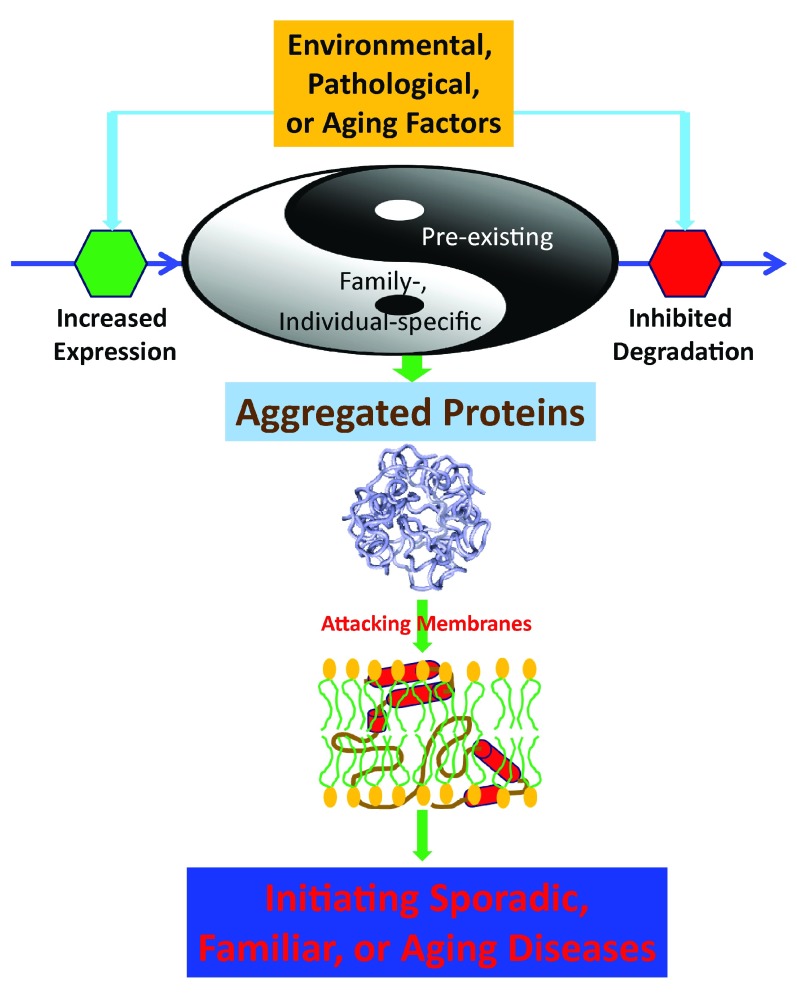
The model for aggregated proteins to initiate sporadic, familiar and aging diseases by commonly attacking membranes. The human genomes appear to contain many pre-existing aggregated proteins like VAPB3. On the other hand, some family members or individuals carry additional genetic mutants like the P56S-VAPB which are insoluble in buffers. Under some environmental, pathological or/and aging conditions, these proteins might be overexpressed, or/and their degradation gets inhibited. As a consequence, the proteins will accumulate and attack membranes to initiate various diseases including aging.

Strikingly, as illustrated in Figure 7, eukaryotic, particularly human, genomes appear to contain many pre-existing proteins like VAPB3, which have no intrinsic ability to fold into well-defined structures and consequently will unavoidably aggregate *in vivo* ^22^. On the other hand, some family members or individuals carry additional genetic mutants like the P56S-VAPB which are insoluble in buffers. Under normal conditions, those proteins are either expressed at low levels, or/and will be removed by degradation machineries such as ubiquitin–proteasome pathway (UPP) and autophagosome–lysosome pathway (ALP). However, triggered by some environmental, pathological or/and aging factors, these proteins might be overexpressed, or/and the degradation machineries get inhibited. As a consequence, the proteins will accumulate and attack membranes to initiate various diseases including aging. Indeed, it has been recently revealed that immediately after synthesis, ~1–2% nascent proteins get degraded in yeast ^87^ while the percentage of proteins that immediately got degraded can dramatically reach ~30% in humans ^88^. Therefore, the homeostasis of various aggregated proteins *in vivo* appears to be the central factor responsible for a variety of human diseases including aging. The number and degree of the membrane attacking by aggregated proteins may serve as an endogenous clock to count down the aging process. Consequently, key approaches to fight against them are to develop strategies and agents: 1) to reduce the expression levels of these proteins; or/and 2) to maintain or/and even enhance the functions of the degradation machineries ^22^; or/and 3) to generate antibodies to clean up the proteins; or/and 4) to design inhibitors to block their interactions with membranes.

## Materials and methods

### Expression and purification of the P56S-MSP domain

The expression and purification of the P56S-MSP domain followed the procedure we reported previously ^26^. Briefly, the expression vectors were transformed into and overexpressed in *Escherichia coli* BL21 (DE3) cells (Novagen). The P56S-MSP protein was only found in inclusion bodies and consequently the pellet was first dissolved in a phosphate buffer (pH 8.5) containing 8 M urea and subsequently purified by a Ni ^2+^-affinity column (Novagen) under denaturing conditions in the presence of 8 M urea. Dithiothreitol (DTT) was then added to the eluted fractions containing P56S-MSP to a final concentration of 100 mM to ensure complete conversion to Cys-SH. After 1 hour, the fractions were acidified by adding 10% acetic acid and subsequently purified by reverse-phase HPLC on a C4 column, and lyophilized.

The generation of the isotope-labeled proteins for NMR studies followed a similar procedure except that the bacteria were grown in M9 medium with the addition of ( ^15^NH _4_) _2_SO _4_ for ^15^N labeling and ( ^15^NH _4_) _2_SO _4_/[ ^13^C]-glucose for ^15^N-/ ^13^C-double labeling ^26^. The purity of the recombinant proteins was checked by SDS–PAGE gels and their molecular weights were verified by a Voyager STR matrix-assisted laser desorption ionization time-of-flight-mass spectrometer (Applied Biosystems). The concentration of protein samples was determined by the UV spectroscopic method in the presence of 8 M urea ^89^.

### Site-directed mutagenesis and spin-labeling

The P56S-MSP domain contains three free Cys residues at positions 41, 53 and 121. As such, the three Cys residues were first mutated to Ala by use of the QuikChange Site-Directed Mutagenesis Kit (Stratagene, La Jolla, CA, USA). Starting from this plasmid, a total of seven single-Cys mutants was prepared: Q6C, D24C, A53C, N68C, M89C, M102C and A121C ( Figure 2B). The mutated plasmids were confirmed by DNA sequencing and their recombinant proteins were subsequently expressed and purified by the same procedures described above. ^1^H- ^15^N heteronuclear single quantum coherence spectroscopy (HSQC) experiments were performed on each mutant to validate that these mutations did not significantly perturb the native structure of the P56S-MSP domain.

The recombinant proteins of seven single-cysteine mutants were Cys-modified following the previous procedure ^33– 35^, by the thiol-reactive nitroxide free radical probe, MTSSL (1-oxyl-2,2,5,5-tetramethyl-∆ ^3^-pyrroline-3-methyl) methanethiosulfonate (Toronto Research Chemicals Inc.). Briefly, the HPLC-purified recombinant protein of the each mutant was dissolved in the buffer containing 8 M urea, 20 mM phosphate (pH 8.0), which was pre-degassed with nitrogen gas for 20 minutes. Subsequently, the MTSSL reagent was added from 3.8 mM stock solution in acetonitrile to reach a ten-fold molar concentration of the protein, followed by incubation at room temperature with constant stirring for 5 hours. To ensure a complete labeling, another dose of MTSSL was added to a ten-fold molar concentration of the protein for an overnight incubation. The MTSSL-labeled protein was purified by reverse-phase HPLC on a C4 column and lyophilized. Based on the verification by the time-of-flight-mass spectrometer, the purity of the MTSSL-modified proteins of all mutants was > 99% after the HPLC purification.

### CD and NMR experiments

All circular dichroism (CD) experiments were performed on a Jasco J-810 spectropolarimeter equipped with a thermal controller using 1-mm path length cuvettes. Data from five independent scans were added and averaged ^26^. The P56S-MSP samples were prepared at a protein concentration of 20 µM in either DMPC vesicles, bicelles formed by DMPC and DHPC, or DPC micelles in water (pH 4.0) and 5 mM phosphate (pH 7.5) respectively.

All NMR experiments were acquired on an 800 MHz Bruker Avance spectrometer equipped with pulse field gradient units as described previously ^47, 48^. NMR data were processed with NMRPipe ^90^ and analysed with NMRView ^91^. For characterizing the conformation of the P56S-MSP in water, a pair of triple-resonance experiments HNCACB, CBCA(CO)NH were collected for the sequential assignment on a ^15^N-/ ^13^C-double labelled sample in 90% H _2_O/10% D _2_O (pH 4.0). For achieving assignments of the P56S MSP domain in DPC micelles, triple-resonance experiments HNCACB, CBCA(CO)NH, HNCO, (H)CC(CO)NH, H(CCO)NH and HCCH-TOCSY were acquired on ^15^N-/ ^13^C-double labelled samples at a protein concentration of 500 µM in protonated DPC micelles (H-DPC) at 100 mM. For obtaining NOE connectivities, ^13^C-edited NOESY experiments were acquired on a double labeled P56S MSP sample in both H-DPC and deuterated DPC (D-DPC) micelles at 100 mM in D _2_O, while ^15^N-edited HSQC-TOCSY and HSQC-NOESY were collected on a ^15^N-labelled sample at a protein concentration of 500 µM in both H-DPC and D-DPC micelles at 100 mM in 90% H _2_O/10% D _2_O.

For assessing the backbone dynamics on the ps-ns time scale, { ^1^H}- ^15^N steady-state NOEs were obtained by recording spectra on the ^15^N-labeled P56S MSP domain at 500 µM in either water or H-DPC micelle (100 mM), with and without ^1^H presaturation with duration of 3 s plus a relaxation delay of 6 s at 800 MHz. To assess conformational exchanges over µs-ms, ^15^N transverse relaxation dispersion experiments were acquired on the P56S-MSP domain in H-DPC micelle, on a Bruker Avance 800 spectrometer with a constant time delay ( *T*_CP_ = 50 ms) and a series of CPMG frequencies, ranging from 40 Hz, 80 Hz, 120 Hz (x2), 160 Hz, 200 Hz, 240 Hz, 320 Hz, 400 Hz, 480 Hz, 560 Hz, 640 Hz, 720 Hz, 800 Hz, and 960 Hz (×2 indicates repetition) as we previously performed ^47, 48^. A reference spectrum without the CPMG block was acquired to calculate the effective transverse relaxation rate by the following equation:

$$R_{2}^{eff}=-1\text{n}\frac{\frac{I\text{(}\nu_{CPMG})}{I_{0}}}{T_{CP}}$$

Where I(ν _CPMG_) is the peak intensity on the difference CPMG frequency and I _0_ is the peak intensity in the reference spectrum.

To probe the orientation of the P56S MSP residues, HSQC spectra of the P56S-MSP in H-DPC were acquired by gradual addition to 10 mM of manganese chloride and gadodiamide (gadolinium(III) 5,8-bis(carboxylatomethyl)-2-[2-(methylamino)-2-oxoethyl]-10-oxo-2,5,8,11-tetraazadodecane-1-carboxylate hydrate) ^35^.

### Generation of NMR constraints and structure calculation

Backbone dihedral angles were generated with TALOS ^+^ by inputting backbone ^1^H, ^15^N and ^13^C chemical shifts ^38^. NOE-based distance constraints were extracted from both ^15^N- and ^13^C-edited NOESY spectra collected on the P56S-MSP samples in D-DPC.

**Figure 8.**
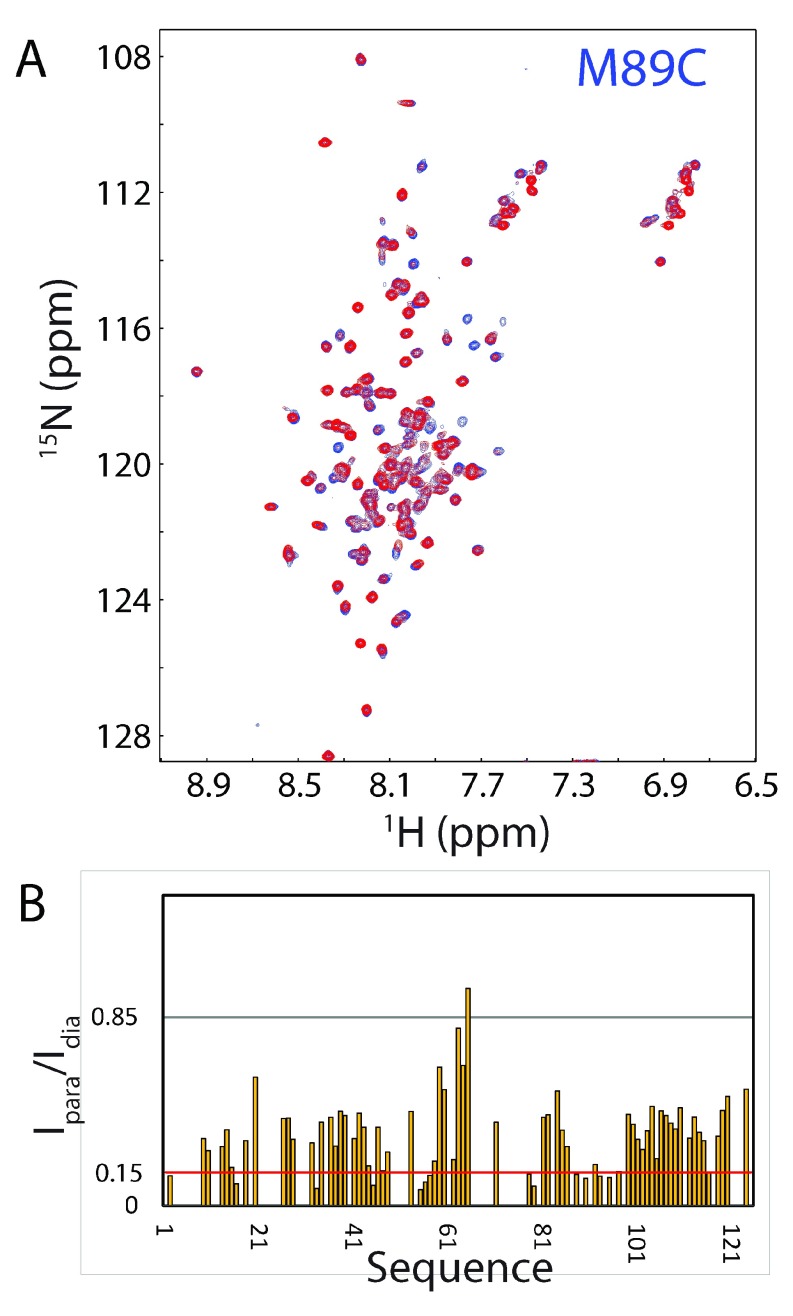
Representative ^15^N HSQC PRE data used to derive long-range distance restraints for structure determination. **A**. Overlay of two NMR HSQC spectra of spin-labeled M89C in the paramagnetic state of the MTSL probe (red) and diamagnetic state after the MTSL probe has been reduced (blue). The HSQC spectra were recorded on a 200 μM ^15^N-labeled M89C sample at 800 MHz and 313 K. **B**. Intensity ratios of HSQC peaks of spin-labeled M89C from the paramagnetic and diamagnetic states.

Paramagnetic relaxation enhancement (PRE) experiments were utilized to obtain long-range distance restraints. Specifically, for each spin-labeled single-cysteine mutant, a pair of 2D ^1^H- ^15^N HSQC spectra were acquired at a protein concentration of 200 µM in 40 mM H-DPC: one for the spin-labeled sample in the paramagnetic form, and another after adding ascorbic acid (to 10 mM) to the sample to reduce the nitroxide, yielding the diamagnetic sample. We also acquired HSQC spectra for 7 corresponding cysteine mutants without spin-labelling at the same conditions and only several HSQC peaks shifted after spin-labeling, indicating that the spin-labeling would not significantly change the conformation. The spectra were subsequently analyzed to obtain PRE-based differences in peak intensities using the programs nmrPipe ^90^ as exemplified by Figure 8 showing the superimposition of HSQC spectra of the M89C mutant in the paramagnetic and diamagnetic forms.

Intensity ratios of peaks from the oxidized and reduced spectra were converted into PREs R2sp by estimating the additional transverse relaxation needed to reduce peak intensity relative to diamagnetic conditions by the observed intensity ratio as previously described ^33– 35^. Peaks unaffected by the paramagnetic probe (intensity ratio > 0.85) were not restrained while peaks with intensity ratio < 0.85 were converted to distances as previously described ^33– 35^. Structure calculations were carried out with the *ab initio* simulated annealing protocol of the Xplor-NIH program and CNS ^36, 37^. The NMR structures of the 125-residue P56S MSP domain in the DPC micelle have been deposited in PDB with ID of 2MDK. Protein structures were analyzed using PROCHECK ^92^ and displayed by PyMol molecular graphics system (W. L. DeLano, DeLano Scientific LLC, San Carlos, CA).
