# Supplementary material for: Resolving the paradox for protein aggregation diseases: NMR structure and dynamics of the membrane-embedded P56S-MSP causing ALS imply a common mechanism for aggregation-prone proteins to attack membranes
Source: F1000Res. 2014 Jul 22;2:221. Originally published 2013 Oct 21. [Version 2] doi: 10.12688/f1000research.2-221.v2 (PMC4168755; doi:10.12688/f1000research.2-221.v2)
Supplement: Peer review history

# Peer review: Referee response for version 2

**DOI:** 10.5256/f1000research.5169.r5545

**Published:** 2014-07-23

**Authors:** H Jane Dyson

**Affiliations:** 1 Department of Molecular Biology, The Scripps Research Institute, La Jolla, CA, USA

The newly revised manuscript is now acceptable.

I have read this submission. I believe that I have an appropriate level of expertise to confirm that it is of an acceptable scientific standard.

# Peer review: Referee response for version 1

**DOI:** 10.5256/f1000research.2665.r2268

**Published:** 2014-07-15

**Authors:** H Jane Dyson

**Affiliations:** 1 Department of Molecular Biology, The Scripps Research Institute, La Jolla, CA, USA

This seems like a very interesting new idea, and the experiments seem well done.

I do find the title a bit confusing. if the proteins are aggregated, how is it that they attack the membranes without needing aggregates? Maybe a qualification such as "a common mechanism for aggregation-prone proteins to attack membranes without the participation of large aggregates"?

The only other small change I would recommend is to check the title of Figure 2. Should this be NMR not NME?

I have read this submission. I believe that I have an appropriate level of expertise to confirm that it is of an acceptable scientific standard.

# Peer review: Referee response for version 1

**DOI:** 10.5256/f1000research.2665.r4030

**Published:** 2014-03-21

**Authors:** Annalisa Pastore

**Affiliations:** 1 National Institute for Medical Research, London, UK

The manuscript by Qin *et al*. describes a structural study of the MSP domain of VAP8 in a membrane-like environment (DPC micelles). The work is overall interesting. However, I have a number of issues that I would like the authors to consider:

1. The title is too general. I do not think that such a generalization is possible at this stage. I think it should be changed into something more relevant for the specific protein.
2. I needed to read down in the introduction to understand which protein it refers to. The VAO8 protein must be cited already in the abstract (and possibly in the title). Some more details on the domain must be given. Is this a beta sandwich? It looks like anIg-like fold. It should be commented on.
3. I do not think the first sentence of the abstract is justified at this level of generalization. I would rewrite it. I am not even sure it is correct. Do the authors refer to the well known problem that toxicity is observed also in the absence of detectable aggregates? It should be clarified.
4. Some of the figures are unnecessary. I would delete figure 5. Figure 6 is unnecessarily large. Most of the figure is unsupported by the data shown in the manuscript. It could be simplified without altering its meaning.
5. The authors should avoid sentences such as: ‘Unexpectedly, five well-formed helices in the membrane environment adopted β-strands in the native MSP fold…’. Why unexpectedly? It is well known that many sequences become helical when in micelles. This has been known for 30 years (my PhD thesis was on a structural study of a peptide in SDS). Likewise, the authors seem to be surprised by observations that have already been reported by others. Without wanting to take anything away from the authors, the idea of a role of poration in neurodegeneration is rather old and has been suggested for Abeta, polyglutamines and several other proteins.
6. Along the logic of 4., the authors should really cite Kagan *et al.* (2012) and Crescenzi *et al*. (2002).

Overall the work is fine and solid but the authors should turn down any claim of generality and/or absolute novelty. The discussion is clear.

I have read this submission. I believe that I have an appropriate level of expertise to confirm that it is of an acceptable scientific standard.
